# Idiopathic degenerative thoracic aneurysms are associated with increased aortic medial amyloid

**DOI:** 10.1080/13506129.2019.1625323

**Published:** 2019-06-18

**Authors:** Hannah A. Davies, Eva Caamaño-Gutiérrez, Ya Hua Chim, Mark Field, Omar Nawaytou, Lorenzo Ressel, Riaz Akhtar, Jillian Madine

**Affiliations:** a Institute of Integrative Biology, University of Liverpool, Liverpool, UK;; b Computational Biology Facility, Technology Directorate, University of Liverpool, Liverpool, UK;; c Department of Mechanical, Materials and Aerospace Engineering, School of Engineering, University of Liverpool, Liverpool, UK;; d Liverpool Heart and Chest Hospital, Liverpool, UK;; e Department of Veterinary Pathology and Public Health, Institute of Veterinary Science, University of Liverpool, Liverpool, UK

**Keywords:** Aortic medial amyloid, medin, thoracic aortic aneurysm, biomechanics, oligomer, bicuspid valve syndrome

## Abstract

**Objective:** To explore the relationship of aortic medial amyloid with biochemical and micromechanical properties of the aortic wall in aneurysm patients.

**Methods:** Human aortic tissues removed during aneurysm surgery from tricuspid (idiopathic degenerative aneurysm, DA) and bicuspid valve (BAV) patients were subjected to oscillatory nanoindentation experiments to determine localised mechanical properties of the tissue (shear storage modulus, *G*´ and shear loss modulus, *G*˝). Collagen, elastin, matrix metalloproteinase 2 and glycosaminoglycans concentrations were determined, along with relative levels of aortic medial amyloid-related factors (medin, milk fat globule-EGF factor 8, oligomers and fibrils). Measurements were combined with clinical data and statistical analyses performed.

**Results:** The DA cohort can be divided based on their phenotype. One group shared similar characteristics with BAV patients, termed bicuspid like phenotype-tricuspid valve. The second group had high amyloid oligomer species present with a significantly lower *G*´ (*p* = .01), indicative of reduced elastic response of the tissue, termed amyloid-rich.

**Conclusions:** We identified a group of DA patients with high amyloid oligomers and altered micromechanical and structural properties of the vessel wall. We propose these findings as a cause for aneurysm formation in these patients. Amyloid is not found in BAV patients, suggesting at least two distinct mechanisms for pathogenesis.

## Introduction

Thoracic aortic aneurysm disease (TAA) is reported to be increasing in frequency with the annual incidence of TAA rupture estimated at approximately 3 per 100,000 population [1]. The incidence will almost certainly increase as the population continues to age.

The aetiology and pathogenesis of TAA is widely varied and not completely understood. Some cases are associated with syndromic connective tissue disorders as a result of mutations in known genes, for example, Marfan’s, Loeys-Dietz and Ehlers-Danlos syndromes. These gene mutations lead to the direct loss of important structural components within the aortic wall that can explain the altered tissue microstructure. Other aetiologies, such as bicuspid aortic valve (BAV) aortopathy, are less understood but efforts have been made to explain the pathogenesis using either a genetic or a haemodynamic model or both. There still remain, however, a large number of cases with non-syndromic sporadic disease and no underlying aetiology. These are currently referred to as idiopathic degenerative aneurysms (DA).

Amyloid fibrils and plaques, made up of over 30 proteins and peptides, are the pathological hallmark of many human disorders [2]. The precise nature of the pathogenic amyloid species is a matter of intense debate. There is increasing evidence that prefibrillar oligomers or intermediates may be the cause in some disorders [3–5].

Aortic medial amyloid (AMA) is estimated to occur in 97% of Caucasians above the age of 50 [6]. The main constituent of AMA is a 50 amino acid polypeptide called medin and is thought to be derived from the proteolysis of milk fat globule-EGF factor 8 protein (MFGE8) [7]. The pathological impact of AMA is unknown, but it is believed that extracellular amyloid accumulation contributes to age-related diminished elasticity of the vessels underlying the pathogenesis of TAA [8]. The potential role of aortic amyloid deposition in BAV aortopathy remains unknown.

It has been documented that expression of matrix metalloproteinase 2 (MMP2) is increased following the addition of medin to cells [8]. Increased MMP2 activity has been observed in TAA and dissection [9]. Patients with BAV also have greater MMP2 activity [10] that correlates with aortic diameter. MMPs have been suggested as diagnostic biomarkers for aortic dilatation in TAA [11]. Furthermore, medial pooling of glycosaminoglycans (GAGs) has been suggested to alter biomechanical properties in thoracic aortic disease [12]. In this study, we investigate the difference in amyloid presence, and associated altered biomechanical and biochemical aortic wall properties, in BAV and DA patients.

## Methods

### Tissue and patient characteristics

Ascending aortic tissue samples were obtained from proximal sections of the aorta during replacement surgery from 26 patients (13 with known bicuspid aortic valves [BAV] and 13 tricuspid aortic valves [designated DA cases]). Patients with known connective tissue disorders were excluded from the study. Tissue was rapidly frozen in super-cooled liquid nitrogen immediately after collection. Patient demographic and clinical characteristics were obtained from the hospital electronic database and recorded. This study was ethically approved by Liverpool Bio-Innovation Hub (project approval reference 15–06), and informed consent was obtained for all participants.

### Biomechanical measurements

Oscillatory nanoindentation was conducted on the tissue samples to determine localised mechanical properties using a KLA-Tencor Nanoindenter G200 with a DCM-II Head (CA, USA), equipped with a 100 µm flat punch indenter (Synton-MDP Ltd., Nidau, Switzerland). For each indentation, the shear storage modulus (*G*´), the shear loss modulus (*G*˝) and the loss factor tan(*δ*), that is, ratio of *G*˝/*G*´ were calculated. The loss factor indicates if the tissue behaves in a more viscous (large tan(*δ*)) or more elastic (small tan(*δ*)) manner. Full methodological details for this oscillatory indentation method can be found elsewhere [13] and summarised in Supporting Information.

### Biochemical measurements

**Figure 1.**
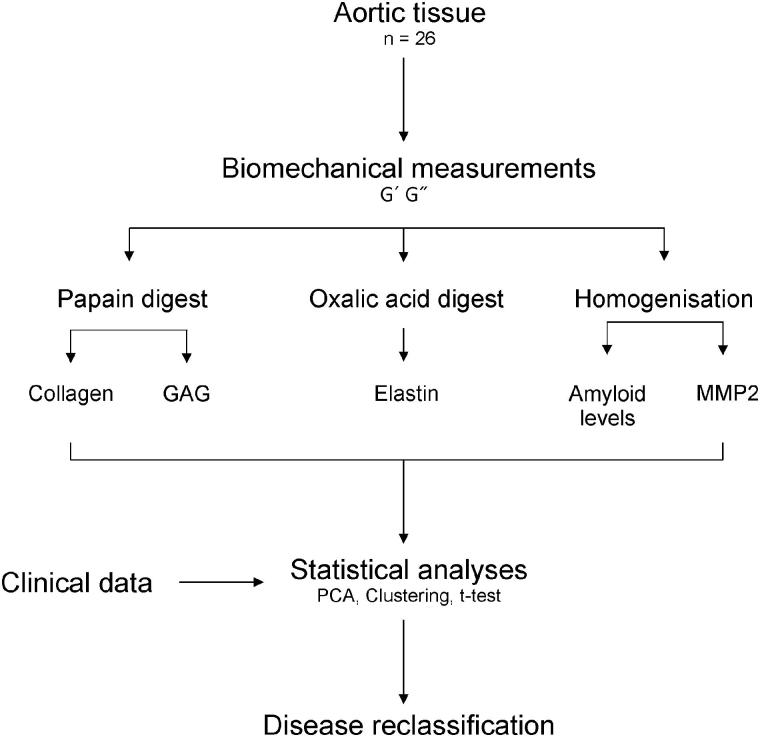
Workflow for the study. Aortic tissue was obtained during aortic replacement surgery from 26 aneurysm patients. Tissue was subjected to biomechanical testing and subsequently digested (using papain or oxalic acid) or homogenised as shown for each biochemical measurement. Biomechanical and biochemical data were combined with patient data to perform statistical analyses which resulted in disease re-classification of the degenerative aneurysm patients into two distinct groups.

Aortic tissue was homogenised or digested with papain or oxalic acid as required (see Figure 1). Collagen content of the tissue was determined by measuring hydroxyproline concentration in the tissue using 1,3-Dimethylbutylamine (DMBA) dye [14,15]. GAG content was measured using dimethyl methylene blue (DMMB) assay [16]. Elastin was measured using Fastin Elastin Kit (Biocolor) according to manufacturer’s instructions. MMP2 ELISA (Thermo) was carried out according to manufacturer’s instructions. Amyloid protein levels were determined by dot blot analysis using primary antibodies from Prothena 18G1 (medin) and 6B3 (MFGE8) [17], or Millipore A11 (oligomers) and OC (fibrils). Blots were analysed using ChemiDoc Imaging System and Image Lab Software (BioRad). Detailed methods are available in Supporting Information.

### Statistical analyses

To test the hypothesis regarding heterogeneity within the DA patients, Ward hierarchical clustering was performed to identify any subgroups within this cohort. Uncertainty of cluster analysis was assessed calculating *p* values via multiscale bootstrap resampling using the package pvclust within the R environment [18]. Clusters with unbiased *p* values larger than 0.90 were considered as true clusters. Further exploration of the data was performed using multivariate transformation principle component analysis (PCA) performed on mean centred and scaled data using the prcomp function within the stats package in the statistical software R [19]. Differences between the identified three groups of patients’ were further assessed via Kruskal-Wallis rank sum test performed for all variables identified as important contributors in the PCA. Pairwise testing of significant variables was performed using the Mann–Whitney–Wilcoxon test. *p* Values were adjusted for false discovery rate via Benjamini and Hochberg method. Detailed methods are available in Supporting Information.

### Histology

Tissues were formalin fixed and paraffin embedded and stained with haematoxylin–eosin (HE) and Verhoeff–van Gieson (EVG). Slides were qualitatively analysed for alteration of the structure of the aortic wall in particular medial degeneration. Cases where evident de-arrangement of elastic fibres and smooth muscle component across the majority of the section area were considered.

## Results

**Figure 2.**
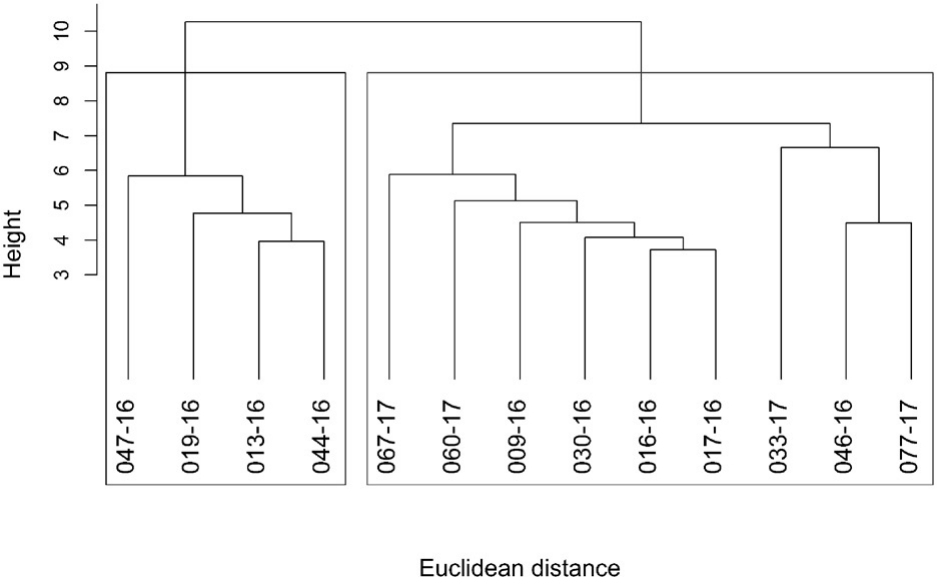
Ward hierarchical clustering of degenerative aneurysm patients. Ward hierarchical clustering for degenerative aneurysm patients (*n* = 13) using Euclidean distance of all the quantitative variables. Boxes represent clusters with an unbiased *p* values over 0.90 indicating that these clusters are robust, thus identifying two groups of patients.

**Figure 3.**
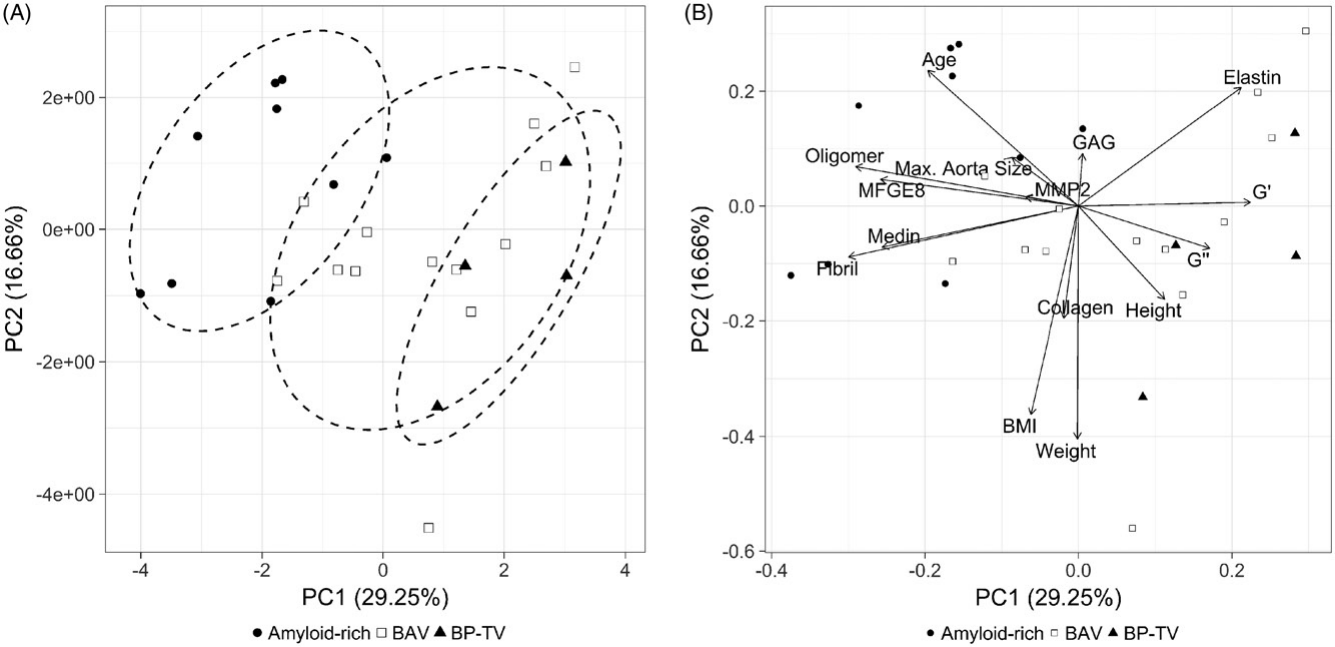
PCA plot with three classification groups. (A) PCA score plot of all quantitative variables for all patients. Each dot represents a patient, which are coloured by their grouping; bicuspid valve (BAV, open squares, *n* = 13), bicuspid like phenotype-tricuspid valve (BP-TV, black triangles, *n* = 4) and amyloid-rich (black circles, *n* = 9). Ellipses represent 75% of the region around the mean of the points of each group. (B) Biplot of the PCA shown in (A) that superposes the scores with the loadings showing that the variables that contribute the most in the separation between the amyloid-rich group and both BAV and BP-TV are oligomers, medin, MFGE8 and fibril levels as well as age, *G*´ and *G*˝.

Biochemical and biomechanical measurements were compiled for all patients (Supporting Information Table S1). In order to identify heterogeneity within the two patient groups, Ward hierarchical clustering was carried out using biomechanical, biochemical and clinical data. Ward hierarchical clustering of BAV patients did not reveal stable clusters at 90% threshold, whereas DA patients resulted in two stable clusters (Figure 2) which pointed towards a highly consistent difference between these two patients’ groups. Further exploration of the data using PCA confirmed the presence of two distinct DA sub-groups. The first two components comprising over 45% of the variance are shown in Figure 3(A). One of the DA patient groups identified in the hierarchical clustering (Figure 3, black triangles) presented high similarity to BAV patients (Figure 3, white squares). On the contrary, the other cluster of DA patients (Figure 3, black circles) did not overlap with the other groups. The loading plot for the PCA (Figure 3(B)), suggested that oligomer, medin, MFGE8 and fibril levels as well as age, and the biomechanical properties, *G*´ and *G*˝ contribute most to the observed separation. Elastin showed a minor contribution, and MMP2, GAG and collagen levels contributed very little to group separation. Based on this analysis we re-classified the DA patients into two distinct sub-groups; bicuspid like phenotype-tricuspid valve patients (BP-TV, black) which share similarity with BAV patients (blue) and amyloid-rich (red).

**Figure 4.**
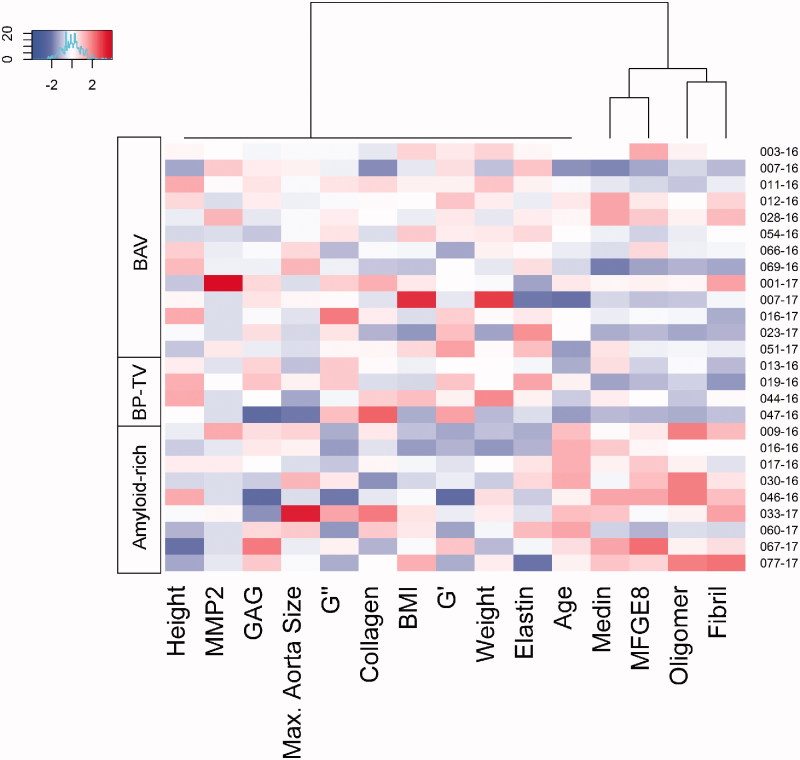
Heatmap of all patients. In rows are shown the patients grouped by their identity as shown. It is noticeable that bicuspid like phenotype-tricuspid valve (BP-TV) share more similarities with bicuspid valve (BAV) patients that amyloid-rich particularly in amyloid characteristics (bottom right corner); medin, MFGE8, oligomer, fibril levels and age. Data was mean centred and scaled. Colours represent lower abundance than the mean (blue), close to the mean (white) and higher abundance than the mean (red). Variables are ordered based on complete-linkage clustering for easy visualisation.

**Figure 5.**
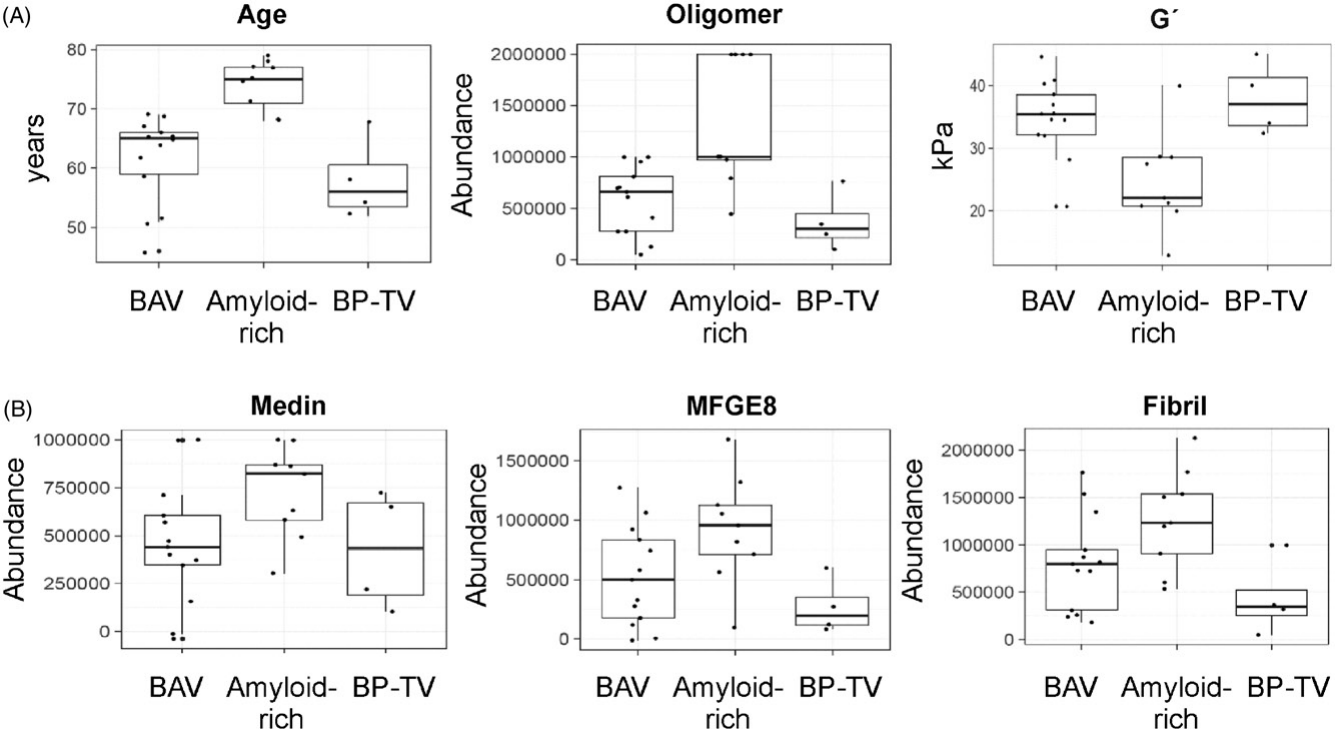
Boxplots of variables between the three groups. (A) Variables that show significant differences between the three groups as shown in Table 1 (bicuspid valve *n* = 13, bicuspid like phenotype-tricuspid valve *n* = 4, amyloid-rich *n* = 9). (B) Additional variables showing increased levels in the amyloid-rich group compared to the other two groups. Variable distribution is shown as boxes containing the interquartile ratio (first and third quantiles) with the median shown (bold line), whiskers represent the 5–95% range, respectively. Each point within the plot represents a patient sample for the variable specified.

**Figure 6.**
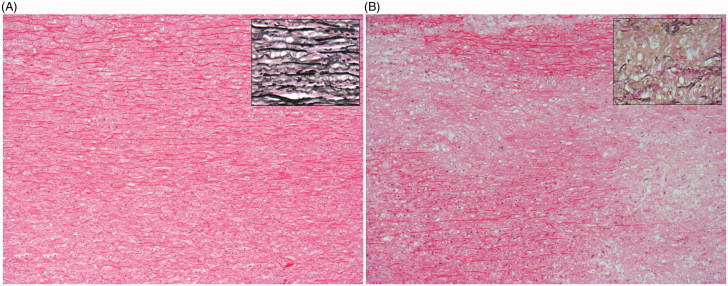
Histological appearance of aortic wall in samples from bicuspid valve (A) and amyloid-rich (B) groups. Appearance of aorta exhibiting a structure within normal limits and correct orientation of elastic fibres (inset) (A). Loss of structure, and de-arrangement of smooth muscle and elastic fibres (inset) (B). Haematoxylin Eosin, original magnification ×100; Inset: Verhoeff-van Gieson (EVG), original magnification ×400.

Further graphical representation as a heatmap (Figure 4) confirmed our hypothesis regarding the role of amyloid-related factors, particularly oligomer level, in this separation. Kruskal–Wallis (nonparametric ANOVA) analysis showed that *G*´ (*p* < .01), oligomer level (*p* < .005), age (*p* < .0005) and *G*˝ (*p* < .05) were significantly varied between the three groups (Supporting Information Table S2, left). Further analysis using Mann–Whitney–Wilcoxon between two groups; BAV and amyloid-rich calculated adjusted *p* values of .01 for *G*´ and oligomer levels and .002 for age (Supporting Information Table S2, right). Boxplots for these variables showed that amyloid-rich patients were older, with increased oligomer levels, and decreased *G*´ (Figure 5(A)). This is consistent with findings above suggesting increased oligomer levels in these patients are likely to be contributing to disease. It should be noted that all patients in this study were above the reported age of 50 for AMA deposition [6]. We also note that despite not showing statistical significance, medin, fibril and MFGE8 levels were also increased in the amyloid-rich group (Figure 5(B)), and observed reduced elastin in these patients (Supporting Information Table S1). Histological analysis of samples exhibited a higher number of cases (5/9) showing marked structural modifications of the aortic wall in the amyloid-rich group compared to BAV (2/13) and BP-TV (1/4; Figure 6).

**Table 1.** Clinical characteristics of patients used in this study.

	All	BAV	DA (all)	BP-TV	Amyloid-rich
	*n* = 26	*n* = 13	*n* = 13	*n* = 4	*n* = 9
Age, years, mean (SD)	65.4 (9.1)	61.5 (7.4)	69.2 (9.2)	58 (7.1)	74.2 (4.2)
Gender					
Female, *n*	13	6	7	1	6
Male, *n*	13	7	6	3	3
Height, cm, mean (SD)	167.9 (10.7)	169.2 (9.9)	166.6 (11.8)	176.3 (7.4)	162.3 (11)
Weight, kg, mean (SD)	80.9 (20.8)	85.2 (22.2)	76.5 (19.3)	84.5 (25.8)	73 (16.1)
BMI, mean (SD)	28.6 (6.6)	29.8 (7.6)	27.4 (5.5)	26.9 (6.5)	27.7 (5.5)
Cholesterol medication, *n* (%)	15 (58%)	6 (46%)	9 (69%)	2 (50%)	7 (78%)
Diabetes	0	0	0	0	0
Steroids, *n*	1	0	1	0	1
Hypertension, *n* (%)	18 (69%)	7 (54%)	11 (85%)	3 (75%)	8 (89%)
Family history aneurysm, *n*	4	2	2	1	1
Smoker (No/Yes), *n*	17 No	9 No	8 No	3 No	5 No
	9 Yes	4 Yes	5 Yes	1 Yes	4 Yes
Aorta diameter, cm mean (SD)	5.2 (1.1)	5.1 (0.6)	5.2 (1.5)	4.1 (1.1)	5.8 (1.4)

BAV: Bicuspid valve; DA: degenerative aneurysm; BP-TV: bicuspid like phenotype-tricuspid valve.

Data are shown as mean (standard deviation, SD) or number of patients (*n*) as shown.

## Discussion

This study has identified that the majority of DA patients display the presence of oligomeric aortic medial amyloid which may play a significant role in TAA pathogenesis. Conversely, this was not observed in BAV patients suggesting that amyloid is not a contributing factor in bicuspid valve-related aortopathy. We suggest that increased oligomer levels in the amyloid-rich DA patients may alter the elastic properties of the tissue and in turn cause dilation of the aorta. However, in the absence of a longitudinal study, we cannot currently confirm a direct cause or effect relationship. In the amyloid-rich group the shear storage modulus (*G*´) which represents the elastic response of the tissue is significantly lower than in the other groups. Low *G*´ values indicate that the tissue is extremely deformable. This group exhibits the lowest tissue stiffness of the three groups. We hypothesise that medin oligomers affect the crosslinking between elastic and collagen fibres within the aorta wall, in turn affecting the normal mechanical response of the tissue which predisposes it to aneurysms and rupture.

We further analysed the predisposition to deformation by looking at the percentage of swelling for each tissue over a 65 min time period. The percentage of swelling was correlated against the micromechanical properties and amyloid levels. We observed the same cluster patterns as shown previously confirming our re-classification of BP-TV patients as BAV-like (Supporting Information Figure S1). Interestingly, amyloid-rich patients exhibited a negative correlation between *G*' and the percentage of swelling, while BAV and BP-TV displayed no correlation (Supporting Information Figure S1(A)). It was also noted that for BAV and BP-TV patients there were no correlations between the percentage of swelling and amyloid levels (Supporting Information Figure S1(B–D)), further confirming our finding that in these patients amyloid is not a contributing factor to disease pathology. In contrast, for amyloid-rich patients, positive correlations were observed between percentage of swelling and all amyloid levels (Supporting Information Figure S1(B–D)). Swelling ratio in hydrogels is a well-defined parameter for determining the mechanical properties of the gels [20], swelling can be altered by the number of crosslinks within a material. As the number of crosslinks within a material increases, the network density increases and thus there will be less room to accommodate fluids within the material, hence swelling would decrease and in turn mechanical properties would increase. The amyloid-rich patients have higher levels of oligomers and fibrils and these aggregated species may disrupt cross-linking within the tissues, therefore these tissues swell more.

Cerebral amyloid angiopathy (CAA) involves deposition of β-amyloid in the walls of small and mid-sized arteries of the cerebral cortex and leptomeninges. Amyloid deposition in CAA has been shown to affect the physiology of the microvasculature and alter the integrity of microvascular responsiveness [21]. Presence of amyloid in CAA is associated with fibrinoid necrosis, fragmentation of vessel walls and has been identified as the site of aneurysms and rupture, with increased amyloid deposition linked to increased aneurysm formation [22]. Vascular amyloid deposition causes structural changes in the walls, weakening vessels through destruction and replacement of the medial layer with amyloid deposits, in turn leading to aneurysm formation through the loss of normal relaxation/constriction capability of the vessel [23]. A statistically significant reduction in *G*´ demonstrates that the stiffness of the amyloid-rich aorta samples has been compromised. This indicates that the structural changes that have previously been reported for CAA also appear to apply in aortic aneurysm formation. Furthermore, *G*˝ was also reduced in the amyloid-rich aneurysm subgroup. *G*˝ is a mechanical property which is particularly sensitive to microstructure [13]. Thus, we hypothesise that the reduced *G*˝ observed in our amyloid-rich aneurysm subgroup may be indicative of altered microstructural changes similar to those observed upon amyloid deposition in cerebral vessels in CAA suggesting a common mechanism of action resulting in aneurysm formation.

Interestingly, there were a higher number of female patients with high amyloid levels (6/9), and the values determined were higher for females than males (1.19E6/0.89E6 for oligomer, 1.13E6/0.87E6 for fibril). The same trend was observed in a previous report for aneurysm patients (0.76/0.56 amyloid, 7.3/5.4 medin) [8]. This suggests a possible female bias for aortic amyloid associated with TAA; however, this requires additional investigation to confirm.

Patient metadata including steroid medication, smoking and family history of aneurysm did not appear to contribute to our analyses as they only affect a small number of patients. Mean BMI values were not significantly different between the groups, indicating that obesity is not a disease related factor in this patient cohort. Hypertension is a risk factor for aneurysm and was found in a high proportion of patients in this study, particularly amyloid-related patients (8/9), with a lower amount associated with BAV (7/13). Similarly, cholesterol medication is a known risk factor and 78% of patients in the amyloid-rich patient group were taking statins, with 46% and 50% in the BAV diagnosed and BP-TV patients, respectively. This suggests that hypertension and high cholesterol could be associated with aortic amyloid-associated disease but this requires further study to confirm. Aorta diameter is below the recommended guidelines for surgery of 5.5 cm in all BP-TV and 85% of BAV diagnosed patients, whereas 44% of amyloid-rich patients have aorta size >5.5 cm. However, it should be noted that aorta diameter was not a major contributing factor to group separation in our analyses.

It is known that aneurysm occurs in BAV patients at a significantly younger age than tri-leaflet patients [24]. Genetics analysis of TAA cases found that patients with a known associated syndrome, for example, BAV, younger age and history of aortic disease display increased risk for carrying a pathogenic or likely pathogenic gene [25]. BAV displays genetic inheritance identified through familial clustering [26,27]. We hypothesise that the BP-TV cohort may have a relative with BAV, and that disease could therefore be due to the presence of a pathogenic or likely-pathogenic gene in these patients.

**Figure 7.**
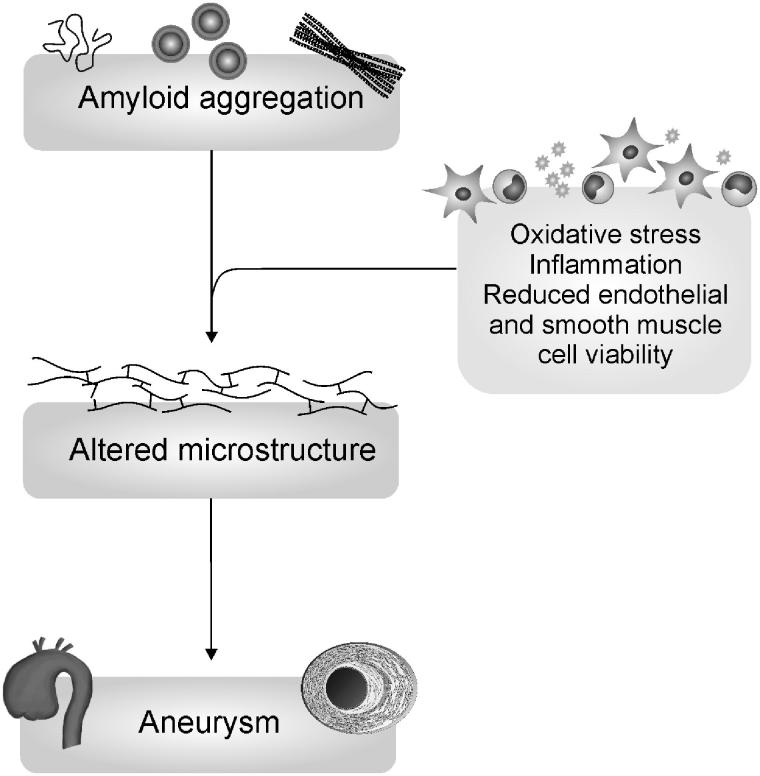
Summary schematic showing hypothesised involvement of amyloid formation in thoracic aortic aneurysm. Data presented here suggests amyloid aggregation (increased oligomers and fibrils) affect the normal mechanical response of the aorta through altered microstructure (decreased *G*´ ) within the aorta wall, in turn leasing to aneurysm formation (grey boxes). It has been shown previously that medin and other amyloid proteins cause enhanced inflammatory and oxidative stress conditions that may provide the link between aggregation and altered aortic wall integrity.

Medin oligomers have been suggested to be associated with TAA; however, this previous study did not measure the oligomer levels instead their conclusions were based on lack of correlation with amyloid fibrils suggesting instead that it was the prefibrillar material that was toxic [8]. To our knowledge, this is the first study to directly show a relationship between oligomeric medin and TAA, as well as a difference in the presence of AMA between BAV and non-BAV aortopathy. Intermediates of medin aggregation have previously been shown to be toxic to smooth muscle and endothelial cells and to promote oxidative stress conditions and inflammatory processes [8,28–30]. These cellular alterations are likely to contribute to altered vessel integrity, in turn causing aneurysm formation. A summary schematic shown in Figure 7 combines our previous knowledge of the effect of medin on cells with current data. We suggest that amyloidogenic species are responsible for altered aortic wall integrity which results in aneurysm development. This represents new mechanistic insights into aneurysm formation in DA patients, further confirms that BAV represents different underling aetiology and provides alternative therapeutic avenues to explore for sporadic TAA.

## Conclusions

This study confirms AMA is increased in a high proportion of DA patients but was not found associated with BAV in our cohort. AMA is associated with altered biomechanical properties and structural modifications of the aortic wall as a likely contributing factor to sporadic aneurysm formation.

## Study limitations

Availability of tissue samples for inclusion in the study results in several limitations that need noting. The first being lack of availability of control patient tissue, preventing correlation with non-diseased patients. In this study we have not probed the microstructural changes using imaging techniques to identify direct links with amyloid. Including greater patient numbers in the study would make the findings more robust, we cannot discard the possibility that by increasing number of BAV patients analysed we might find an amyloid-rich group of patients. Future work will include study of larger number of patients to try to address this question. Finally, as the tissue is removed during surgery we are unable to correlate any parameters measured in this study with onset of disease.

## Supplementary Material

Supplementary_Information.docx

## Abbreviations

AMA: aortic medial amyloid
BAV: bicuspid aortic valve
BP-TV: bicuspid like phenotype-tricuspid valve patients
CAA: cerebral amyloid angiopathy
DA: degenerative aneurysm
DMBA: 1,3-dimethylbutylamine
DMMB: dimethyl methylene blue
EVG: verhoeff–van Gieson
GAG: glycosaminoglycan
HE: haematoxylin–eosin
MFGE8: milk fat globule-EGF factor 8
MMP: matrix metalloproteinase
PCA: principle component analysis
TAA: thoracic aortic aneurysm
