# Supplementary material for: Idiopathic degenerative thoracic aneurysms are associated with increased aortic medial amyloid
Source: Amyloid. 2019 Jun 18;26(3):148–55. doi: 10.1080/13506129.2019.1625323 (PMC6816484; doi:10.1080/13506129.2019.1625323)
Supplement: Supplementary_Information.docx [file IAMY_A_1625323_SM1550.docx]

**Supplementary Information**

# Idiopathic degenerative thoracic aneurysms are associated with increased aortic medial amyloid

^a^Hannah A. Davies,  ^b^Eva Caamaño-Gutiérrez, ^c^Ya Hua Chim, ^d^Mark Field, ^d^Omar Nawaytou, ^e^Lorenzo Ressel, ^c^Riaz Akhtar, ^a*^Jillian Madine

**Table S1: Biomechanical and biochemical measurements for all patients in this study.** Bicuspid valve (BAV), degenerative aneurysm (DA), bicuspid like phenotype-tricuspid valve (BP-TV).

|  | All  n=26 | BAV  n=13 | DA (all)  n=13 | BP-TV  n=4 | amyloid-rich n=9 |
| --- | --- | --- | --- | --- | --- |
| G´ (kPa) | 32.6 (8.1) | 35.9 (5.6) | 29.3 (9) | 38 (5) | 25.4 (7.7) |
| G˝ (kPa) | 13.2 (3.5) | 14 (2.7) | 12.3 (4.1) | 15.6 (2.2) | 10.9 (4) |
| GAG (µg/mg) | 3.1 (1) | 3.2 (0.5) | 3.1 (1.4) | 3.0 (1.6) | 3.1 (1.4) |
| Collagen (µg/mg) | 39.1 (9.7) | 37.1 (7.7) | 41.1 (11.2) | 45.8 (12.4) | 39 (10.7) |
| Elastin (µg/mg) | 61.9 (18.7) | 65.9 (18.6) | 57.9 (18.8) | 66.2 (16.8) | 54.2 (19.3) |
| MMP-2 (ng/ml) | 78.4 (124.9) | 107.5 (158.9) | 49.2 (73.5) | 25.3 (38.6) | 59.9 (84.5) |
| medin level (10E5) | 5.5 (3.1) | 4.6 (3.2) | 6.4 (2.9) | 4.3 (3.1) | 7.3 (2.4) |
| MFGE8 level (10E5) | 6.2 (4.6) | 5.2 (4.2) | 7.2 (5) | 2.7 (2.4) | 9.3 (4.6) |
| oligomer level (10E5) | 8.2 (6) | 5.8 (3.3) | 10.5 (7.2) | 3.7 (2.8) | 13.6 (6.3) |
| fibril level (10E5) | 9.1 (5.6) | 8.1 (5) | 10.1 (6.2) | 4.3 (4) | 12.7 (5.3) |

Data is shown as mean (SD). GAG, collagen and elastin were measured as µg/mg of wet tissue. MMP-2 was measured as µg/ml of homogenised tissue. Medin, MFGE8, oligomer and fibril level were determined by analysis of dot blot using Image Lab (Bio-Rad) using adjusted volume values.

**Table S2: Results from Kruskal-Wallis rank test for three patient groups (bicuspid valve, bicuspid like phenotype-tricuspid valve and amyloid-rich) and Mann-Whitney-Wilcoxon test between two patient groups (bicuspid valve and amyloid-rich).**

|  | Kruskal-Wallis rank test between three groups | Mann-Whitney-Wilcoxon test between bicuspid valve and amyloid-rich patients | | |
| --- | --- | --- | --- | --- |
|  | p-value | 95% confidence interval | raw p-value | adjusted p-value |
| G' | **0.0068** | (4.604,16.473) | **0.0056** | **0.0111** |
| G'' | 0.0417 | (0.19,6.52) | 0.0434 | 0.0694 |
| Fibril | 0.0663 | (-970089.144,118250) | 0.0708 | 0.0708 |
| Oligomer | **0.0043** | (-1304157,-180721) | **0.0048** | **0.0111** |
| Medin | 0.0563 | (-527422,0) | 0.0608 | 0.0708 |
| MFG8 | 0.0663 | (-827221.328,19557.542) | 0.0708 | 0.0708 |
| Age | **0.0003** | (-17,-6) | **0.0003** | **0.0024** |

Significant variables after p-value adjustment G´, oligomer level and age are shown in bold.

**Supplementary Methods**

**Biomechanical Measurements**

Oscillatory nanoindentation was conducted on the tissue samples to determine localised mechanical properties of the tissue. The tests were conducted using a KLA-Tencor Nanoindenter G200 with a DCM-II Head (CA, United States), equipped with a 100 µm flat punch indenter (Synton-MDP Ltd., Nidau, Switzerland). Three biopsy strips at a thickness approximately 0.5 cm were taken from each patient sample. Sixteen indents were made on the medial layer of each sample with a spacing of 200 µm. All tests were conducted at 110 Hz with a pre-compression of 7 µm applied to the tissue. Prior to indentation, the samples were hydrated in phosphate buffered saline (PBS) and placed in a custom-designed liquid cell. Samples were mounted under the nanoindenter and sectioned in transverse. Hence, the indenter tip (which is always perpendicular to the sample) was tested on the central region of the medial layer. All testing was completed within 64 mins after de-thawing the samples. All measurements were conducted at room temperature (22^o^C). For each indentation, the shear storage modulus (G´), the shear loss modulus (G˝) and the loss factor tan (δ) i.e. ratio of G˝/ G´ were calculated. The loss factor indicates if the tissue behaves in a more viscous (large tan(δ)) or more elastic (small tan(δ)) manner. After each indent, the tip was cleaned by indenting a piece of double-sided Scotch tape (3M, Atherstone, United Kingdom) mounted on an adjacent sample puck before returning to the tissue sample. Full methodological details for this oscillatory indentation method can be found elsewhere [1].

**Swelling ratios**

Images were taken of tissues before and after PBS uptake over a period of 65 mins. The area of each tissue was measured using FiJi and normalised against the area of the holder. The percentage of swelling was calculated using the difference between the area of the tissues before and after PBS uptake.

**Biochemical Measurements**

**Tissue preparation**

10 % w/v aorta homogenates were prepared in PBS, using 5x30sec homogenisation cycle in Precellys CK28 tubes using a MiniLys®.

Papain digestion: Papain was used at 10units/ml in 0.1M sodium acetate, 2.4mM EDTA, 5mM L-cysteine, pH 5.8. 50mg tissue was incubated in 500µl of papain solution at 60°C overnight (until completely digested).

Oxalic acid digestion: 750µl of 0.25M oxalic acid was added to 7.5mg of tissue and heated at 100°C for 1 h. Samples were allowed to cool and centrifuged at 21,000 x g for 10 min. The supernatant was removed and digestion repeated a further 4 times with fresh oxalic acid. 200µl from each digestion was pooled (total 1ml).

**Collagen**

Hydroxyproline concentration is measured to approximate the collagen content of the tissue [2, 3]. 100µl of 12M hydrochloric acid was added to 100µl of papain digested tissue and autoclaved. Following this samples were lyophilised and re-dissolved in 2ml ddH_2_O. 50µl of hydroxyproline standard was added to 200µl ddH_2_O prior to addition of 250µl of diluent (2:1 propan-2-ol:ddH_2_O). 250µl of oxidant (0.42g chloroamine T, 5ml ddH2O, 25ml of stock buffer containing (6.87g sodium acetate, 7.5g trisodium citrate.2H_2_O, 1.1g citric acid, 80ml propan-2-ol made up to 200ml with ddH_2_O)) was added and left for 20 mins at room temperature. 250µl of colour reagent (3g 1,3-Dimethylbutylamine (DMBA), 4.5ml 70% perchloric acid, 25ml propan-2-ol) was added and left for 14 mins at room temperature followed by heating at 70°C for 20 mins. Samples were left to cool for 10 mins before reading the absorbance at 550nm. Hydroxyproline is present in collagen at 14%, so this value is used to calculate the collagen content of the tissue compared to a standard curve. Samples were analysed in triplicate with 3 biological replicates.

**Glycosaminoglycans**

Papain digested aorta samples were diluted 1 in 10 with water and added to a 96 well flat-bottomed transparent plate in a volume of 40μl. 250μl of dimethyl methylene blue (DMMB) dye (16mg 1-9 dimethyl methylene blue, 2g sodium formate, 2ml formic acid, in 1 litre water, pH 3.5) was added to each well and absorbance at 570nm read immediately compared to a standard curve of Chondroitin Sulphate C [4]. Samples were analysed in triplicate with 3 biological replicates.

**Elastin**

Elastin was measured using Fastin Elastin Kit (Biocolor) according to manufacturer’s instructions using 200µl of pooled oxalic acid digests in duplicate with 3 biological replicates.

**Amyloid**

10µL of homogenised tissue was dotted onto nitrocellulose membrane and allowed to dry. Membranes were blocked in 5% normal goat serum in PBS for 1h at room temp on a shaker. Membranes were incubated with primary antibody overnight (prothena 18G1 medin, prothena 6B3 MFGE8 [5], Millipore A11 amyloid oligomers, Millipore OC amyloid fibrils) at 1:1,000 in PBS containing 0.05% Tween (PBS-T) with rotation at 4°C. Membranes were washed with PBS-T (3x15mins) prior to addition of secondary antibody (StarBright700, BioRad, 1:20,000) for 1 h at room temperature with agitation. A further 3x15mins PBS-T wash steps were carried out prior to visualisation on a BioRad ChemiDoc Imaging System. Blots were analysed using Image Lab Software (BioRad) with auto-exposure.

**MMP2**

MMP2 ELISA (Thermo) was carried out according to manufacturer’s instructions using 50µl of aorta homogenate (10% in PBS) in duplicate with 2 biological replicates.

### Statistical Analyses

### Hierarchical clustering

DA patients’ data was used to calculate the Euclidean distance. These data were clustered via Ward method and the uncertainty in the hierarchical cluster analysis was assessed using the package pvclust [6] within the R package that uses multiscale bootstrapping to assess the hypothesis on whether a cluster does exist. Bootstrapping was performed with n=1000. Clusters with an unbiased p-value larger than 0.90 were considered as true clusters and taken forward in the analysis.

### Principal component analysis

Data structure was further assessed with the multivariate transformation PCA which was performed on mean centred and scaled data using the prcomp function within the stats package in the statistical software R [7]. All graphical representations were done using the package ggplot2 [8].

### Univariate testing

The differences between the three groups of patients’ i.e. BAV, BP-TV and amyloid-rich were further assessed via Kruskal-Wallis rank sum test performed for all variables identified as important contributors in the PCA. Post hoc testing was performed using Mann-Whitney-Wilcoxon test by comparing the variables between the BAV patients and the DA patients classified as amyloid-rich. P-values were adjusted for false discovery rate via Benjamini and Hochberg method.


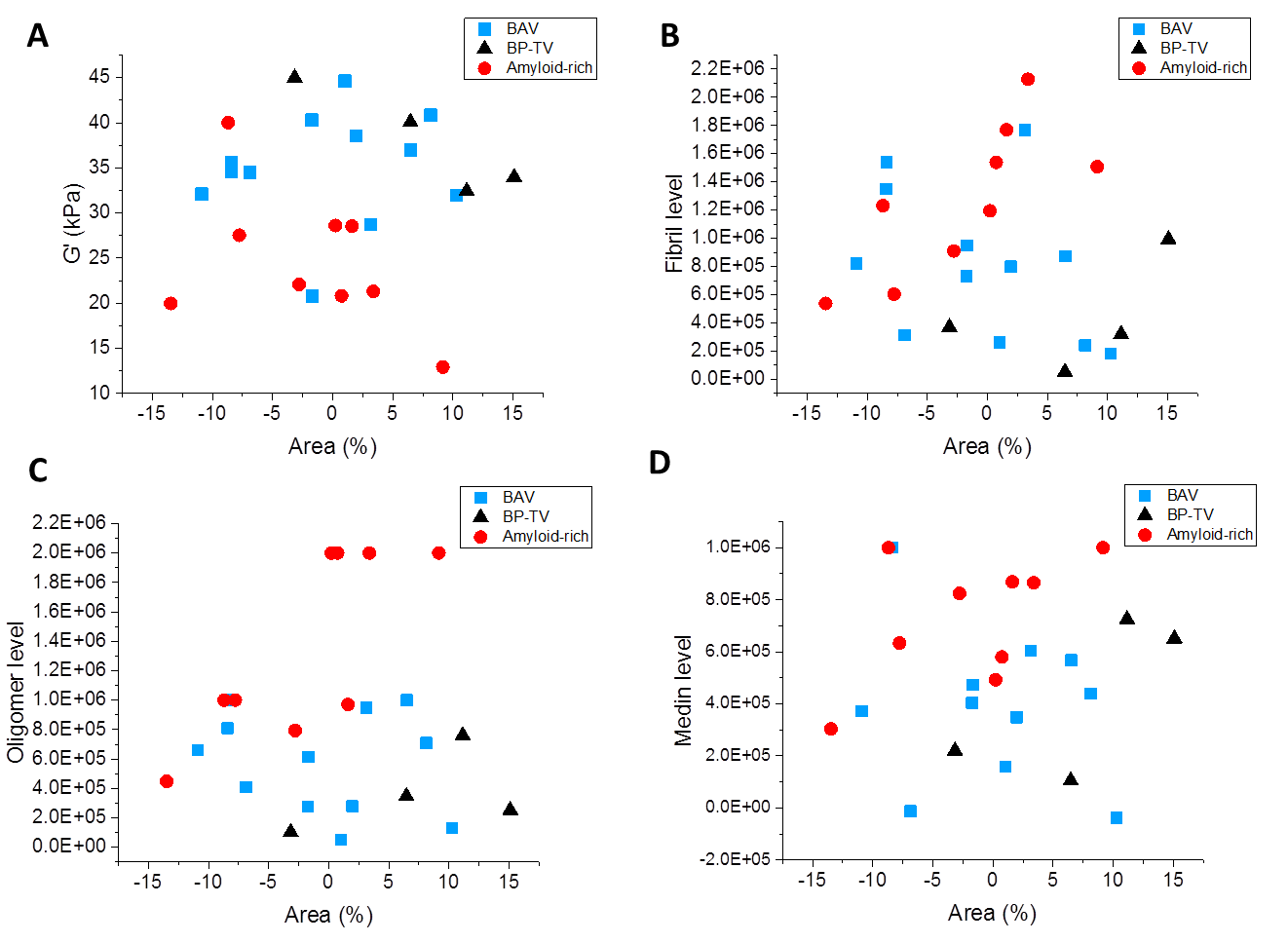


**Figure S1: Swelling ratios of aortic tissue following exposure to PBS for 65 minutes.** Swelling is expressed as percentage change in area of tissue and shown correlated with G´ (A), fibril level (B), oligomer level (C) and medin level (D) for each patient coloured according to their sub-group (bicuspid valve, bicuspid like phenotype-tricuspid valve and amyloid-rich) as shown.

**Supplementary References**

1. Akhtar R, Draper ER, Adams DJ, et al. Oscillatory nanoindentation of highly compliant hydrogels: A critical comparative analysis with rheometry. J Mater Res. 2018;33(8):873-883.

2. Bannister DW, Burns AB. Adaptation of the Bergman and Loxley technique for hydroxyproline determination to the autoanalyzer and its use in determining plasma hydroxyproline in the domestic fowl. The Analyst. 1970 Jun;95(131):596-600.

3. Bergman I, Loxley R. Two improved and simplified methods for the spectrophotometric determination of hydroxyproline. Anal Chem. 1963 1963/11/01;35(12):1961-1965.

4. Farndale RW, Sayers CA, Barrett AJ. A direct spectrophotometric microassay for sulfated glycosaminoglycans in cartilage cultures. Connect Tissue Res. 1982;9(4):247-8.

5. Shughrue P, Alexander L, Higaki JN, et al. Novel anti-medin antibodies detect medin deposits in aortic aneurysm, marfan syndrome and other cardiovascular diseases. 15th International Symposium on Amyloidosis. 2016;Abstract #25252.

6. Suzuki R, Shimodaira H. Pvclust: an R package for assessing the uncertainty in hierarchical clustering. Bioinform. 2006 Jun 15;22(12):1540-2.

7. Team RC. R: A language and environment for statistical computing. R Foundation for Statistical Computing, Vienna, Austria 2017;<https://www.R-project.org/>.

8. Wickham H. ggplot2: Elegant Graphics for Data Analysis. Springer-Verlag New York 2016.
